# Effect of Fungal Deterioration on Physical and Mechanical Properties of Hemp and Flax Natural Fiber Composites

**DOI:** 10.3390/ma10111252

**Published:** 2017-10-31

**Authors:** Bryn Crawford, Sepideh Pakpour, Negin Kazemian, John Klironomos, Karen Stoeffler, Denis Rho, Johanne Denault, Abbas S. Milani

**Affiliations:** 1 Composites Research Network-Okanagan Node, School of Engineering, University of British Columbia, Kelowna, BC V1V 1V7, Canada; bryn.crawford@ubc.ca; 2 Department of Biological Engineering, Massachusetts Institute of Technology, Cambridge, MA 02142, USA; spakpour@mit.edu; 3 Department of Biology, University of British Columbia, Kelowna, BC V1V 1V7, Canada; kazemiannegin@hotmail.com (N.K.); john.klironomos@ubc.ca (J.K.); 4 National Research Council-Automotive and Surface Transportation, Boucherville, QC J4B 6Y4, Canada; Karen.Stoeffler@cnrc-nrc.gc.ca (K.S.); johannedenault@icloud.com (J.D.); 5 National Research Council-Aquatic and Crop Resource Development, Montréal, QC H4P 2R2, Canada; Denis.Rho@cnrc-nrc.gc.ca

**Keywords:** biocomposites, hemp and flax natural fibers, fungal deterioration, material properties, design considerations

## Abstract

The development and application of bio-sourced composites have been gaining wide attention, yet their deterioration due to the growth of ubiquitous microorganisms during storage/manufacturing/in-service phases is still not fully understood for optimum material selection and design purposes. In this study, samples of non-woven flax fibers, hemp fibers, and mats made of co-mingled randomly-oriented flax or hemp fiber (50%) and polypropylene fiber (50%) were subjected to 28 days of exposure to (i) no water-no fungi, (ii) water only and (iii) water along with the *Chaetomium globosum* fungus. Biocomposite samples were measured for weight loss over time, to observe the rate of fungal growth and the respiration of cellulose components in the fibers. Tensile testing was conducted to measure mechanical properties of the composite samples under different configurations. Scanning electron microscopy was employed to visualize fungal hyphal growth on the natural fibers, as well as to observe the fracture planes and failure modes of the biocomposite samples. Results showed that fungal growth significantly affects the dry mass as well as the tensile elastic modulus of the tested natural fiber mats and composites, and the effect depends on both the type and the length scale of fibers, as well as the exposure condition and time.

## 1. Introduction

Over the past decades, fiber-reinforced polymer composites (FRPC) have received increased attention in a variety of industries ranging from aerospace to ground transportation and sporting equipment [1]. These materials offer desired advantages with respect to performance, for example, improved strength-to-weight and stiffness-to-weight ratios, over classical metallic materials [2]. They also present lower processing costs as the final product form and properties are developed concurrently, resulting in a high degree of production tailorability [3]. In 2011, the world market value of end-use products manufactured from composites was reported to be USD $55.6 billion and the North American composites industry grew at approximately 9% annually [4]. This strong growth is expected to continue in coming years, driven by factors including falling prices of carbon fiber, uptake of emerging material technologies such as natural fibers and the introduction of industry-led research and development (R&D) activities focused on innovation. To further exemplify, the overall Americas market for advanced composites in wind energy alone has been expected to triple to USD $25.8 billion by 2020 [4].

Concerning natural fiber reinforced composites (NFRC), their global market reached $2.1 billion in 2010, with a compound annual growth rate of 15% in the five subsequent years [5]. A significant contributor to this growth is the global industrial appetite for application of biocomposite constituents ranging from bio-sourced polymer resins, plant-based fibrous materials as reinforcement, nano-cellulose fillers, among other material forms. Among recently developed NFRCs, the hemp, flax, ramie, kenaf, sisal and jute natural fibers are among the most commonly utilized [6,7,8]. Manufacturing composite products from such plant-based precursors have attracted attention also due to the growing incentives by governments for developing greener products and innovative technologies, in an effort to reduce the widespread dependence on fossil fuels [9,10,11] and to move toward more recyclable, biocompatible, and biodegradable products [12,13,14,15,16]. In light of this, eco-friendly bio-composites from natural fibers (NF) are perceived as offering significant added value for the environment and economy [12]. Most plant-derived fibers are made up of varying amounts of principle components including cellulose, hemicellulose, and lignin that affect fibers’ mechanical and physical properties [13,14], and in turn depend on factors such as retting level, plant species, genealogy and epigenetic factors.

In NFRC, the natural fibers provide the bulk of the laminate strength and stiffness, whereas the matrix material offers high degrees of tailorability with respect to other required properties in design; for example, polypropylene (PP) as a polymer matrix in a biocomposite can offer desired properties such as potential transparency, dimensional stability, flame resistance, high impact strength, in addition to resistance to various forms of degradation [5,8], depending on the chemical formulation of the bulk polymer process. Presently, NFRC applications are seen in prominent and large-scale industry sectors such as construction, automotive, marine, and electronics [6,9,10], at both structural and non-structural levels. However, their consideration by designers is still associated with disadvantages such as high moisture absorption under moist environmental conditions, quality variations, and susceptibility to photodegradation, sunlight, insects, and microbial growth [5,13]. These sources of material degradation or failure are not typically within the bounds of modeling or testing of classical composites engineering, leaving an ongoing gap in knowledge and hindering confidence in wider implementation of emerging NFRC materials and associated manufacturing technologies. Theoretically, microorganisms have the potential to interact with natural components of NFRC during many stages of their lifecycle. This includes harvesting, processing, spinning, weaving and finishing of the raw fibers, transportation and storage, manufacturing into laminates, and even in situ when subjected to moist environmental conditions (e.g., in marine applications) [14,17]. Microorganisms can grow on natural fibers and deteriorate them by producing extracellular enzymes such as hydrolytic enzymes (e.g., cellulase, lignase, etc.) [18,19,20,21]. For this reason, maintenance of physical and mechanical properties of NFRC at a satisfactory level needs to be considered by designers and manufacturers.

This study attempts to better understand the nature of microorganism-based biodeterioration of NFRC. It is postulated that natural fibers, once harvested and processed, will still contain spores of various fungal species, which under proper environmental conditions of moisture and temperature can proliferate and lead to material properties deterioration. Similarly, during processing and/or in-service, more fungal species may be introduced and increase the rate and magnitude of deterioration. To mimic the latter, *Chaetomium globosum*, which is the most common fungus isolated on wet cellulosic building materials and found in both outdoor and indoor environments [14,22], has been selected. In regards to design applications, it is also of particular interest to assess how different types of natural fibers, for example, hemp and flax, would differ in their relative rates of biodeterioration.

## 2. Materials and Methods

### 2.1. Material Selection and Preparation

Flax and hemp straws were subjected to the so-called field or dew-retting process, which is known to improve the decortication process yield, but more importantly it allows the recovery of the bundles of cellulosic fibers located directly under the cuticle [13]. Natural fibers (NF; flax and hemp fibers) were obtained from an advanced decortication/cleaning process developed by the National Research Council of Canada (NRC). These two NF fibers were then comingled with polypropylene fibers (PP, by American Synthetic Fiber, Pendergrass, GA, USA), at a weight ratio of 1:1, and turned into none-woven mats by Texel (Texel Technical Materials Inc., Saint-Elzéar, QC, Canada). These two (50%–50%) NF-PP mats along with the two chop strand mats, made of 100% flax and 100% hemp fibers and fabricated by Tekle Technical Services Inc., Edmonton, AB, Canada, were used to conduct the current investigation (i.e., with a total of four material types).

The material specifications of the natural fibers in the selected mats are outlined in Table 1; it should be noted that the compositions listed do not necessarily indicate those for all hemp and flax plants in consideration as reinforcement in biocomposites, as significant batch-to-batch and breed-to-breed differences should be expected for these natural materials. Upon receiving the test materials, they were cut into 80 mm × 40 mm specimen sizes using a paper guillotine blade. The guillotine provided a clear edge cut of the NF mats and NF-PP mats due to the tight tolerance between the cutting edges and stiffness of the assembly. The specimens were subsequently placed individually in sterile dry Petri dishes for further processing as follows.

### 2.2. Fungal Spore Suspension

*Chaetomium globosum* was chosen as the fungal species to inoculate the four mat samples. This species is one of the most common fungi isolated on damped cellulosic building materials. It can be found in both outdoor and indoor environments [14,22]. Pure cultures of multiple strains of isolated *C. globosum* were ordered from the University of Alberta Microfungus Collection and Herbarium (UAMH, Edmonton, AB, Canada). The strains were isolated from indoor environment, likely similar to those present in indoor material storage facilities and composite manufacturing sites. *C. globosum* was cultured on slants of Difco potato dextrose agar (PDA) at room temperature (25 °C), until confluent growth and sporulation were achieved. Spore suspensions containing 10^5^ spores/mL were prepared using sterile water [23].

### 2.3. Experimental Design

From a microbiology point of view, to investigate the effects of indigenous fungi (if any) versus that of inoculated *C. globosum* fungus, three environmental conditions were tested: (i) no water or fungal spores was added; (ii) water (2 mL) was added but no fungal spores was added; and (iii) water (2 mL) and fungal spores were both added. The first subgroup mimics an ideal storage conditions, which is a dry environment with only few native (indigenous) fungal species embedded into the raw material; the second subgroup mimics those environmental conditions favorable (i.e., high relative humidity) to the growth of indigenous microbial species; then, the third subgroup mimics the same environmental conditions as in (ii) but at a high fungal loading rate. The later was obtained by inoculating 2 mL of a *C. globosum* spore suspension (105 spore/mL). All plates were stored in a growth chamber and incubated at 25 °C for 7-day (t_7_) and 28-day (t_28_) time periods. In all sample plates, a smaller plastic container filled with sterile water was placed, mimicking a damped storage condition as it can be observed in industrial practice. Each week, sterile water was added into the respective plates to ensure the maintenance of a high relative humidity level and hence continuous fungal growth.

#### 2.3.1. Physical Testing

All four type of samples were tested for weight loss, as the primary indicator for the breakdown and degradation of fibrous material. These tests were performed at the time intervals of t_0_, t_7_, and t_28_, each repeated four times. At each time point, samples were oven dried at 65 °C for a duration of 7 days prior to dry mass determination.

#### 2.3.2. Mechanical Testing

At each time point, 15 plates containing NF-PP mat samples were removed from Petri dishes, and consolidated using a Carver Auto Series NE Automatic #4393-ASTM Hydraulic Press (Carver Inc, Wabash, IN, USA). Samples were laminated as single-ply composite sheet (laminae) under a pressure of approximately 320 N per sample. This was performed at a temperature of 190 °C, applied with a ramp-rate of 10 °C/min from room temperature, for a hold time of 80 mines to ensure proper consolidation, before cooling to room temperature. The cooldown rate was regulated through a compressed air and water cooling system, such that the development of variation in residual stresses in the samples could be minimized and be consistent across the samples. Laminated composite samples were cut into dog-bone tensile specimens using an abrasive waterjet (AWJ) cutting-Omax 2652 JetMachining Centre (Omax, WA, USA). Samples were inspected for edge quality to prevent premature failure from defects and conditioned to 23 °C and 50% relative humidity for at least 24 h prior to mechanical testing. Samples were tested in a universal tensile machine (Instron 5969 load frame with a 50 kN load cell (Instron, Norwood, MA, USA), using a wedge-action jaw fixture. The tests were performed as a modified version of ASTM E8, where the modification was made to account for the constraints imposed on the sample dimensions of 80 mm × 40 mm due to the Petri dish and incubation equipment. The tensile tests yielded the load-history of the samples from which their Young’s modulus (stiffness) could be calculated using engineering stress and strain values. The calculated stiffness variations were used for subsequent analyses on the effect of natural fiber material types against simulated environmental conditions and associated fungal deteriorations.

#### 2.3.3. Scanning Electron Microscopy

For the visualization of microstructures, as well as the fungal mycelia and spores on NF mats and NF-PP mats, before (t_0_) and after (t_28_) physical/mechanical testing, a Tescan Mira3 XMU Field Emission Scanning Electron Microscope/SEM (Tescan, Kohoutovice, Česká republika) was employed. Fungal colonies grown on the water-fungi group were also identified using a standard optical microscope.

## 3. Results

### 3.1. Physical Testing

**Figure 1:**
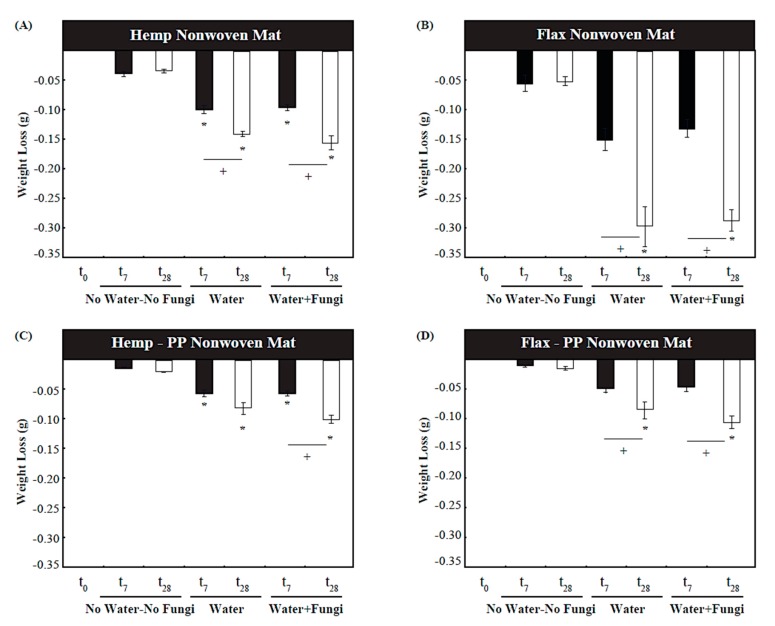
Weight loss testing for the four natural fiber mat types, each subjected to three different exposure conditions, measured at periods of 0, 7 and 28 days; the “*” sign indicates statistical significance (with a *p*-value < 0.05) compared to the control sample at t_0_ (i.e., with no mass loss). The “**+**” signs indicate significate within the sample groups (i.e., the effect of exposure time). (**A**) Hemp Nonwoven Mat; (**B**) Flax Nonwoven Mat; (**C**) Hemp–PP Nonwoven Mat; (**D**) Flax-PP Nonwoven Mat.

The mass of the mat samples was taken over a 28-day experimental period and at least four repeats of each measurement were made. Results (Figure 1) show the average mass loss for each sample group. It is noted that within each group, as time progresses, there has been a net reduction in the natural fiber mat mass. This is to be expected, as the process of respiration is performed by fungi metabolizes cellulosic matter, converting the more complex and longer organic molecules into water, carbon dioxide and other fluid species, which egress from the samples. When compared to initial condition (i.e., at t_0_), the maximum weight losses under the extreme condition of water-fungi were observed for the four materials types as follows: (i)—28.58% flax mat (F-1401); (ii)—15.40% hemp mat (H-1416); (iii)—10.82% for flax-PP mat (F-1375), and; (iv)—9.80% hemp-PP mat (H-1381), all showing a statistical significance level of *p* < 0.05. The former two sample groups are made of only NF, whereas the latter two groups have been made of 50% NF, and consequently the observed weight losses are proportionate to the NF content.

Of particular interest, a mass loss as high as 28.58% was also seen at t_28_ for samples subjected to water only (i.e., with no inoculation with the spore suspension). This supports the hypothesis that there are indigenous fungal species colonizing the test material, deposited during previous stages of the fibers’ lifecycle. Such fungi likely establish when the plants are in their natural environment, when they are harvested and processed in heavy machinery, dried, or shipped and otherwise handled throughout the supply chain. Fungal growths and hyphae were visually observed on these samples with naked eye. Also using standard optical microscope, it was noticed that *Chaetomium* sp. was present in the as-received material, among other fungal or bacterial taxa. It is assumed that there would be limited mass loss due to chemical species dissolving into the water surrounding the sample, as the cellulose and hemicellulose that comprises the fibers, as well as other longer chain polymers and inorganic species, are water-insoluble [18,19]. Hence, such a significant change in mass (~1% per day) of the latter group of samples must have stemmed from other mechanisms such as respiration by consumption of the biochemical species by fungi.

### 3.2. Mechanical Testing

**Figure 2:**
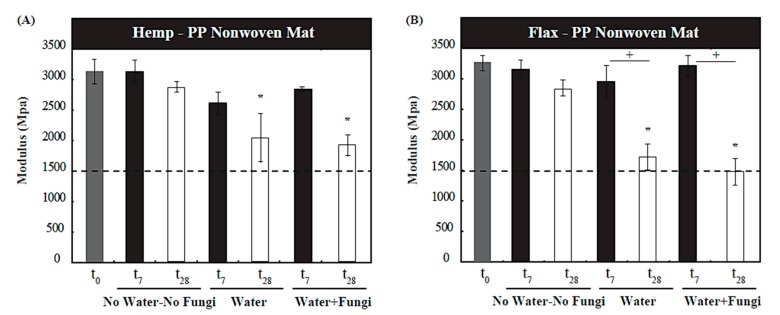
Young’s modulus of (**A**) hemp and (**B**) flax NFRC samples at 0, 7 and 28 days. Samples in columns with a “*” were significantly different from the baseline (control at t_0_) with a *p*-value < 0.05. The dashed line represents typical modulus of elasticity for the same grade polypropylene used in the NFRC specimens (approximately 1500 MPa). The “**+**” signs indicate significance within the sample groups (i.e., the effect of exposure time).

From the stress-strain responses collected from mechanical tests, the Young’s moduli of consolidated composite samples were calculated; it is of note that consolidation and subsequent mechanical tests were only meaningful for the samples consisting of a matrix (in this case, PP). Figure 2 illustrates the mechanical test results, comparing the hemp-PP and flax-PP laminates at t_0_ (preconditioned control samples), t_7_ and t_28_. Results demonstrate that the stiffness of control samples (with no water or fungi) have remained statistically unchanged after 28 days of incubation. However, for the hemp NFRC samples inoculated with sterile water (the second environmental group) and those with additional fungal spore suspensions (the third environmental group), after 28 days show a significant decrease in Young’s modulus at 33.84% (*p* < 0.05) and 37.96% (*p* < 0.05), respectively. Similarly, for the flax NFRC samples, those inoculated with the sterile water (the second group) and those with additional fungal spore suspensions (the third group), after 28 days show a sizeable decrease in Young’s modulus at 46.09% (*p* < 0.05) and 53.91% (*p* < 0.05), respectively. The control samples of both hemp and flax NFRCs showed similar Young’s moduli, approximately 3.0–3.3 GPa, illustrating both the similarity of these natural materials as the mechanical reinforcement, as well as the matrix-dominant properties of the composite when using a volume fraction of 0.5 and a randomly-oriented reinforcement [2].

Theoretically, there are several factors that influence the Young’s modulus of composites, including the chemical compatibility between the fibers and matrix constituents, the processing conditions used in the cure (thermoset) or crystallization (thermoplastic) of the laminate, the properties of the matrix material, the dispersion, architecture and concentration of fibers, as well as the individual fiber properties and internal morphology [23,24,25,26,27,28]. Given that the PP matrix, its surface treatment and volume fraction was common among all the laminated samples, it was not deemed to be the main contributing factor to the observed differences between different NFRC types tested. The degradation of modulus is more pronounced in the flax NFRC samples after 28 days, compared to the hemp NFRC samples. This would also suggest that the flax fibers are potentially more susceptible to degradation by fungal species, as was also noticed through the weight loss analysis in Section 3.1. An interesting observation of the results in Figure 2 was also that over time, the Young’s modulus of both hemp and flax composite samples decreased and approached to the modulus of PP matrix alone (approximately 1500 MPa), which would suggest a significant degradation of the natural fibers and their bonding with the matrix.

**Figure 3:**
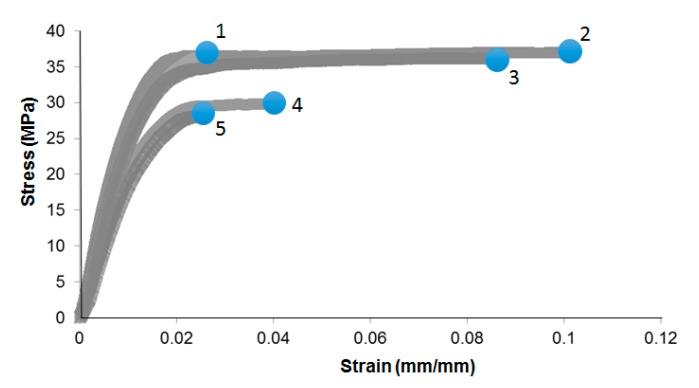
Repeats of engineering stress-strain response of a typical natural fiber reinforced composites (NFRC) sample tested under tensile loading; notice non-repeatability of the material behavior beyond the linear elastic limit, in particular the maximum elongation at the failure point (represented by a circle for each curve), as well the corresponding tensile strength.

It may be hypothesized that biodegradation of the natural fibers should also affect the tensile strength and energy absorption capacity of the NFRC, due to loss of fibers mass and possibly the reduced fiber-matrix interface strength [29,30]. The latter would be through the phenomena of “equilibrium toughness” and “progressive damage” [27] whereby the level of adhesion between the dissimilar materials is altered, lowering the threshold for decoupling and ultimately, mechanical failure. To assess, it was attempted to calculate the values of tensile strength and energy absorption through the engineering stress-strain curves for each group of the laminates. However, in the case of selected nonwoven NFRCs, the strain to failure varied by a significant amount (Figure 3), while the response in the elastic regime remained fairly repeatable. The bridging of fibers across a fracture plane that can retain loads, as well as the slow process of fiber pull-out, are seemingly random phenomena, especially given the length scale of the randomly chopped fibers themselves and as such, the principles of classical micromechanics for long-fiber composites is not valid. In light of the observed large discrepancy (non-repeatability) of failure points (Figure 3), no statistical analysis could be performed on tensile strength and toughness properties of the sample groups. However, on average, it was noted that at a given period of environmental exposure, there was a monotonic decreasing trend of these properties between the samples exposed to no water or fungi and those exposed to the water and/or fungal spore suspension. It is known that the mechanical properties of chop strand mat reinforced biocomposites highly depend on the cellulose content and microfibrillar angles [31], which would have been significantly affected by the presence of fungal species in the tested samples.

### 3.3. Scanning Electron Microscopy

SEM analysis was performed on select samples in order to extract visual information regarding (i) the level of fungal growth on the natural fibers, which could be observed from the presence of hyphae on the natural fibers of unconsolidated mats, and (ii) the effect they had on micromechanics of NFRCs by examining the fracture surfaces of the consolidated samples after being loaded to tensile failure.

**Figure 4:**
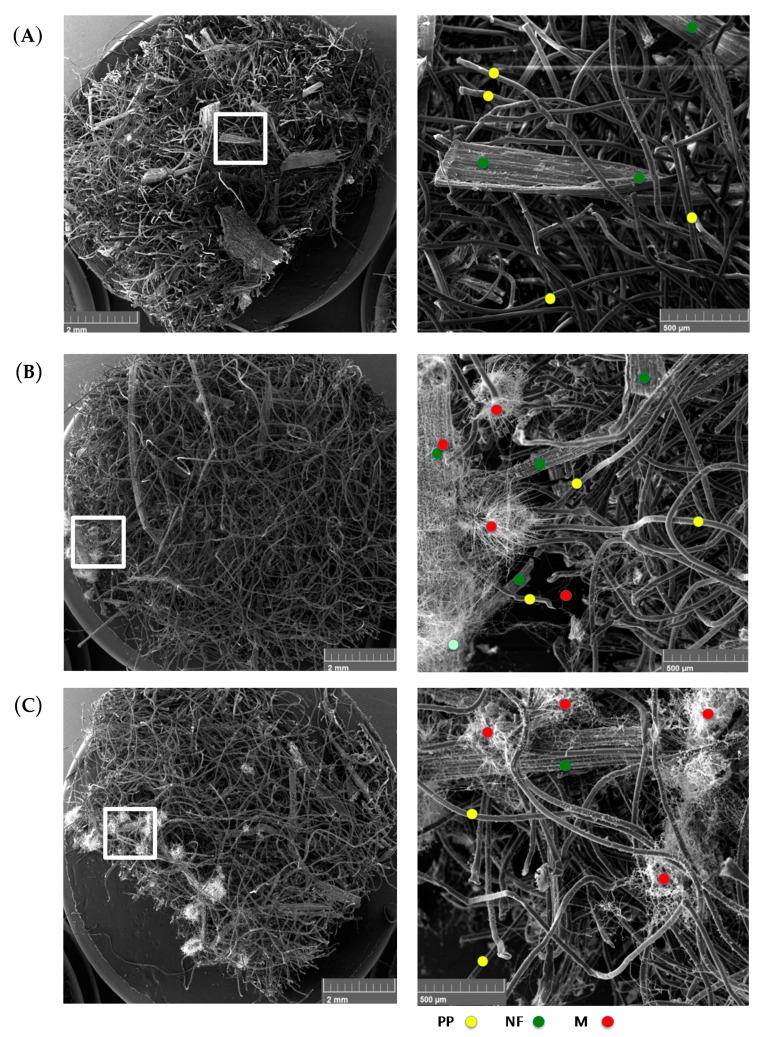
Scanning electronic microscope (SEM) micrographs with low (left) and high (right) magnification for the hemp-PP dry mats at t_28_, under different exposure conditions. The conditions included (**A**) no water-no fungi, (**B**) yes water-no fungi, and (**C**) yes water-yes fungi. It is noticed that fungi grew on the natural fibers under wet ambient conditions, i.e., without the addition of any fungi, as in (**B**), indicating that there have been omnipresent fungal species in the material prior to its processing. Notice the extent and distribution of fungal growth in (**C**). Yellow, green and red circles indicate the presence of sample PP fibers, natural fibers, and mycelium fungus.

**Figure 5:**
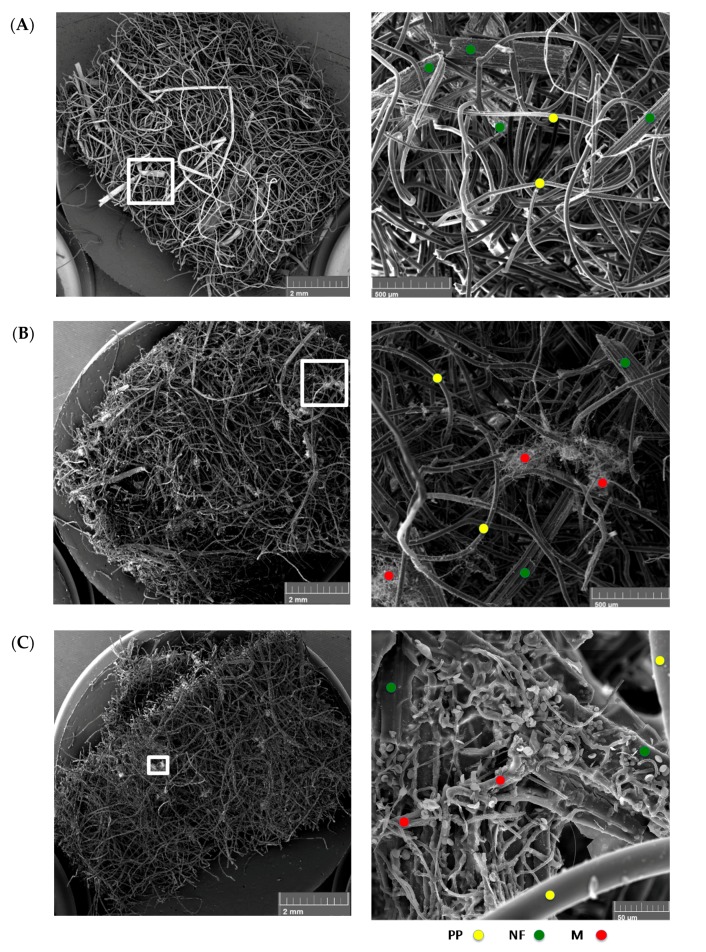
SEM micrographs with low (left) and high (right) magnification for the flax-PP dry mats at t_28_ under different exposure conditions. The conditions included (**A**) no water-no fungi, (**B**) yes water-no fungi, and (**C**) yes water-yes fungi. Similar to the hemp fiber composite, here (**B**) indicated that there have been omnipresent fungal species in the NFRC prior to its processing. Yellow, green and red circles indicate the presence of sample PP fibers, natural fibers, and mycelium fungus. Also notice the clear presence and extent of fungal spores (white dots) in the 25X magnified image (**C**).

Images were taken at two magnifications for both hemp-PP (Figure 4) and flax-PP (Figure 5) dry mats after 28 days of incubation to allow for the greatest level of fungal growths. The SEM micrographs (Figures 4A and Figure 5A) clearly show the non-homogeneity in the NF-PP composites, where the large particles seen are the natural fibers and the thin, much more regular and tubular structures are the polypropylene fibers. With the addition of water in the 28-day exposure of the material, (Figures 4B and Figure 5B), fungal growth was clearly identifiable. This suggests that the as-received mats, without any further conditioning to inoculate them with fungal spores, must have already contained some fungal species. This conjecture supports the earlier quantitative results in Section 3.1 and the hypothesis that natural fiber mats can be prone to attack from fungal species under a broader set of conditions than originally anticipated. Under the extreme condition, when the samples are exposed to fungi and water (Figure 4C and Figure 5C), both the hemp and flax NFRC mats showed significantly higher level of fungal growth. An additional note of interest was that even handling during cutting the edges of the specimens by scissors or the paper guillotine for SEM preparation, could have the potential to impact the fugal growth distribution; as evidenced from low magnification images (Figure 4 and Figure 5) by more fungal growths towards the exterior edges of the samples. This in turn would have an additional implication for manufacturers or end users: cleaned or sterilized tools may be highly necessary while both producing and utilizing these materials in their lifecycle.

**Figure 6:**
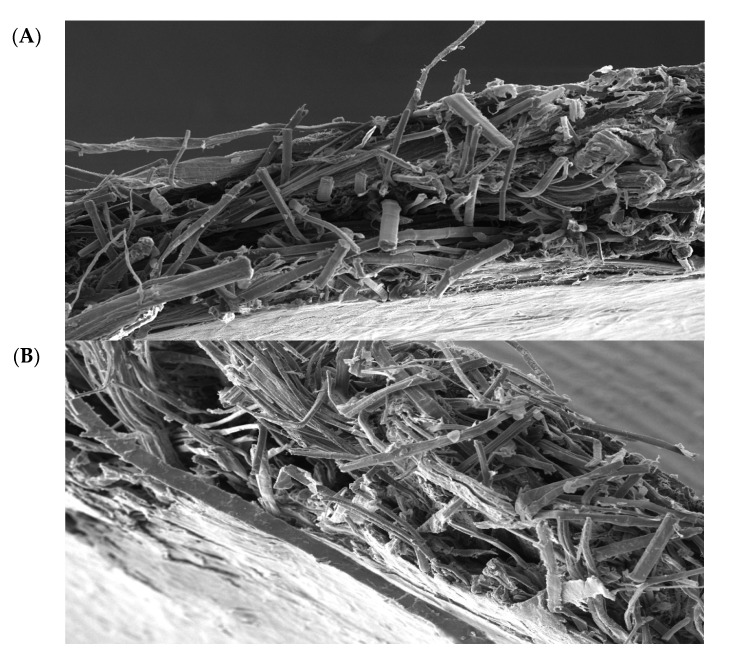

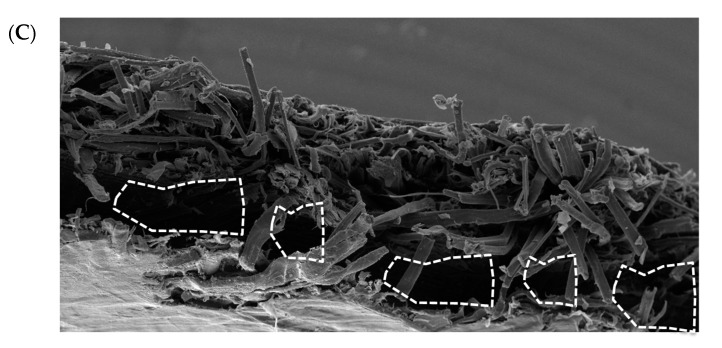
High-magnification SEM micrographs of fracture planes of laminated flax-PP fiber mats, under different exposure conditions following the 28-day incubation time period. The conditions included (**A**) no water-no fungi, (**B**) yes water-no fungi, and (**C**) yes water-yes fungi. In (**A**), there are observed regions of higher PP concentration on the outer faces of the sample. In the case of (**B**), there are some instances of fiber pull-out, as indicated by the more local bunching of the fibers and larger relative gaps between the bunches. For the condition (**C**), fiber pull-out appears to be much more prominent, with larger observable gaps, to the point where delamination between the outer PP-rich layer and inner fiber-rich layer has occurred (shown as hatched areas).

It is worth noting that the high degree of variability seen in the SEM images with respect to microstructure and distribution of the natural and PP fibers, may be viewed an epistemic nature of mat-reinforced biocomposites and, in turn, can lead to a high level of non-repeatability with respect to mechanical response of the composite samples, as was also noted from mechanical tests beyond the elastic limit (Figure 3). Excessive microscopic flaws in the composite mat structures, such as fibers curling or kinking in shape, are geometric factors of instability that can contribute to premature failures during repeats of mechanical testing of composites. At the consolidated level, in short-fiber laminated composites under tensile loading, shear deformation in the matrix is known to be a significant factor to the fibers’ maximum load bearing capacity (fibers shorter than a critical length cannot carry maximum loads) as well as to the subsequent initiation and propagation of damage within the composite [24]. Accordingly, further SEM imaging was performed on the consolidated flax-PP samples, under the three exposure conditions, without removing any of the previously seen fungal growths in the dry mats. The fracture plane of the no water-no fungi condition is shown in Figure 6A. It can be seen that there are distinct discontinuities in the material microstructure, leading to a heterogeneous fracture plane under tensile loading. The natural fibers are embedded within the PP matrix, yet there is a higher concentration of PP towards the two outer faces of the sample, which is inherent to the closed molding manufacturing process. Figure 6B represents the water-exposed condition and Figure 6C the water- and fungi-exposed condition. Similar to Figure 6A, a high degree of discontinuity in the fracture planes in Figure 6B,C is observed. However, some of the failure modes appear more exacerbated by the fungal growths observed on the samples prior to consolidation. Namely, in the case of Figure 6B, there are some regions of fiber pullout, which indicate a premature disbanding of the fiber and matrix constituents. This supports the hypothesis that the fungal growth may affect the surface conditions of the fibers. In Figure 6C, fiber pull-out is even more prominent and there is evidence of delamination between the outer polymer layer and the inner material, which was not nearly as pronounced in the other fracture planes. This, once again, implies that the greater degree of fungal growth has caused more severe failure modes within the NFRC.

## 4. Discussion: A Material Selection Perspective

Having shown the effect of fungal deterioration on mechanical properties of different NFRC, a more design-specific question may be posed: What are the underlying factors for observed differences in property degradation between the flax and hemp composite samples? Accordingly, which type of mats should be considered more biodegradation resistant for design purposes?

Based on the results in Figure 1A,B, the tested 100% flax natural fiber samples lost a greater relative mass than their counterpart hemp natural fiber samples. This difference has been deemed statistically significant as the magnitude has been in the order of three to four times. It is believed that this observation is the result of two underlying factors. First, flax has a higher cellulose content, as well as a lower hemicellulose content, when compared to hemp. Namely, per Table 1, by mass, the 100% flax samples (Mat F-1401) contained 73.8% cellulose and 13% hemicellulose, while the 100% hemp samples (Mat H-1416) contained 72.8% cellulose and 14% hemicellulose. Cellulose is a more readily available carbon source for microbial fungi [32,33,34] and can be preferentially and rapidly consumed as compared to hemicellulose. Hence, the higher relative cellulose content of the flax fibers is expected to lose mass faster than the hemp fibers. As a second factor, the flax fibers has had a much larger surface area (Table 1; also Figure 4 and Figure 5), which would offer a higher number of sites for the fungal enzymes. Similarly, the flax-PP samples (Mat F-1375) contained higher cellulose (69.8%) and less hemicellulos (14.2%) as compared to the hemp-PP samples (Mat H-1381), which contained 67.2% cellulose and 15.7% hemicellulose. On the other hand, concerning the second underlying factor, the average fiber surface area in the flax-PP samples (247 × 10^3^ μm^2^) has been less than that of the hemp-PP samples (928.3 × 10^3^ μm^2^). Subsequently, there trade-off between these two factors has resulted in a slight increase of mass loss of the flax-PP composite compared to its hemp based counterpart (Figure 1C,D). Interestingly, the relative mass loss for the 100% hemp and 50% hemp-50% PP samples have been in the same order of magnitude (Figure 1), whereas this difference for the case of flax samples is quite pronounced (the 100% flax sample lost much greater fraction of mass compared to 50% flax-50% PP sample), which again may be directly explained through the corresponding values of aforementioned factors.

Regarding the material mechanical behavior, given the same volume fraction of matrix, random nature of fibers distribution, and comparable fiber aspect ratios (length to dimeter), the Young’s moduli of the two tested hemp and flax NFRCs have been similar before deterioration takes place (Figure 2). However, upon exposure and deterioration, the flax based samples have yielded lower mechanical property, owing to the same factors discussed above for the mass loss. In addition, comparing Figure 1 and Figure 2, it is noticeable that the relative rate of deterioration of modulus has been higher than that of mass, which can be explained by the fact that a change in fiber dimension has a proportional relation with mass but a non-linear proportion with effective elastic properties in chop strand mat composites, through affecting the interfacial strength parameters [26]. Overall, it appears that under identical matrix and environmental exposure conditions, the tested hemp NFRC samples are more resistant to the microbial deterioration of composite properties.

## 5. Conclusions

There is growing interest in higher technology industries, such as automotive and marine, to use composites derived from renewable resources. Natural fibers used as a reinforcement constituent have been strong candidates for this purpose. However, the effect of biodegradation of such biocomposites is an ongoing concern, especially when they are subjected to moisture and damp environmental conditions during storage, manufacturing, or in service. When raw natural fibers are exposed to high relative humidity, potential premature structural failure can occur. Both the hemp and flax fibers that were the focus of this study, which were reinforced with polypropylene as a thermoplastic matrix, showed clear signs of biodegradation. Fungal communities were seen with the naked eye and under the microscopy. The probabilistic nature of fiber distribution in the mat-based NFRC materials is believed to be a source of uncertainty and variability that outstrips the contribution of the fungal growths on the timescales observed. It was shown that longer exposure time (28 days) could potentially lead to significant changes in both physical (e.g., mass) and mechanical (e.g., Young’s modulus) of the materials.

An important observation was that the NFRC host (indigenous) fungal species can grow and consume the natural materials under, for example, humid storage conditions. This effect was particularly seen in the weight loss tests, where a statistically significant change in this physical property (about 15% for 100% hemp mat, and 30% for 100% flax mat) were observed over time when samples were placed in sterile water (i.e., with no addition of *C. globosum* subjectively). Likewise, this concurred with results seen in the Young’s modulus tests for the same group of samples (about 33% for hemp based composite, and 43% for the flax-based composite). Observations made using SEM imaging further supported this indication, where fungal hyphae were noted on samples that were not inoculated with fungal spores. Through the 100% natural fiber and the 50% natural fiber-50% PP mats tested, it was expected that the samples with a greater presence of food sources (i.e., biodegradable) for fungal growth should lose relative mass and mechanical properties faster. This hypothesis was supported through analyzing two underlying factors:

(i) Higher percentage of cellulose (less lignin) in the natural fibers; and
(ii) Higher surface area (diameter and length) of the embedded natural fibers.

These factors should be of consideration for designers and manufacturers using NFRC, especially when considering different types of natural fiber mats for material selection purposes. These factors would similarly have implications on the choice of storage and/or operation conditions. Humidity will provide an environment for omnipresent fungi on the materials to proliferate and consume and degrade the natural fibers. This, in conjunction with the consideration of fiber forms and relative rates of fungal consumption, can determine the useful shelf life of dry (unconsolidated) NFRC, as well as guide the development of standards and procedures to maximize their durability upon consolidation and use in service. Overall, for the tested samples, it was noted that the hemp NFRCs are more resistant to the microbial deterioration of material properties as compared to their flax counterpart. Of course, this observation should also be justified in the view of higher price of hemp in the market than flax, despite hemp crop’s much higher yield throughout different seasons [35].

Further investigation into this field is deemed necessary before complete standards and procedures can be developed for reliable use of natural fibers in engineering applications under indoor/outdoor humid conditions. In particular, it is recommended that further study be pursued on the correlation between different microbial communities present on NFRC, the type of fibers, and the processing conditions applied during harvesting, extraction, and surface treatments [36], and with the aim to eventually better understand the nature of biological processes underlying microbial deterioration of different properties in the final products. Different antimicrobial chemicals can be studied as an additive on NF to prevent the propagation of fungi.

## Figures and Tables

**Table 1:** Specifications of the natural fibers within the tested nonwoven mats. Polypropylene (PP).

Chemical Composition & Geometrical Features	Mat Type			
	H-1381 (Hemp-PP)	F-1375 (Flax-PP)	H-1416 (Hemp)	F-1401 (Flax)
Cellulose (%)	67.2	69.8	72.8	73.8
Hemicellulose (%)	15.7	14.2	14.0	13.0
Lignin (%)	13.5	11.7	10.3	10.3
Shive (%)	5.8	17.1	10.8	10.4
Fiber diameter (μm)	39.4	22.5	32.1	29.9
Fiber length (mm)	5–10	2–5	2–5	10–15
Fiber surface area (μm^2^)	928.3 × 10^3^	247.4 × 10^3^	353.0 × 10^3^	1174.2 × 10^3^
